# Cranioplasty complications and risk factors associated with bone flap resorption

**DOI:** 10.1186/s13049-015-0155-6

**Published:** 2015-10-06

**Authors:** Tor Brommeland, Pål Nicolay Rydning, Are Hugo Pripp, Eirik Helseth

**Affiliations:** Neurosurgical Department, Oslo University Hospital Ullevål, Po Box 4950 Nydalen, Oslo, Norway; Oslo Centre of Biostatistics and Epidemiology, Research Support Services, Oslo University Hospital, Po Box 4950 Nydalen, Oslo, Norway; Faculty of Medicine, University of Oslo, Oslo, Norway

**Keywords:** Craniectomy, Cranioplasty, Bone flap resorption, Post-operative complications

## Abstract

**Background:**

Decompressive craniectomy (DC) may be performed in patients with acutely raised intracranial pressure due to traumatic brain injury or stroke. It is later followed by a cranioplasty procedure (CP) in the surviving patients. This procedure is associated with a high frequency of post-operative complications. Identifying risk factors for these adverse events is important in order to improve the clinical outcome. This study examines possible predictive parameters for post-operative complications in CP.

**Methods:**

Retrospective, single institution review of all patients undergoing a DC for acutely raised intracranial pressure over a 10 year period at Oslo University Hospital Ullevål, Norway. Subsequent CP using autologous bone flaps or synthetic implants were registered along with all post-operative complications. Predictors of post-operative complications were identified using uni –and multivariable regression analyses.

**Results:**

A DC was carried out in 125 patients, of whom 33 died, 4 were lost to follow-up, and 1 (an infant) later underwent cranial remodeling. A CP was performed in the remaining 87 patients. Post-operative complications were recorded in 31 (36 %) patients of whom 22 lost their primary implant. Surgical site infection (SSI) and bone flap resorption (BFR) were the two most common complications, affecting 8 (9.2 %) and 14 (19.7 %) patients, respectively. Only BFR was associated with some of the recorded variables. Using multivariable logistic regression analysis, young age (OR = 0.94, 95 % CI 0.88-1.00, *p* = 0.04), bone flap fragmentation (OR = 14.3, 95 % CI 2.26-89, *p* = 0.005), long storage time (OR = 1.03, 95 % CI 1.00-1.04, *p* = 0.02) and Glasgow Outcome Scale at the time of cranioplasty (OR = 2.55, 95 % CI 1.04-6.23, *p* = 0.04) were found to be significant risk factors for bone flap resorption.

**Conclusions:**

Cranioplasty after decompressive craniectomy carries a high rate of complications. In this study, SSI and BFR were the two most common complications of which predictive clinical parameters could be identified for BFR only. The results indicate that synthetic implants may be considered in pediatric patients and in cases with fragmented bone flaps or delayed time to cranioplasty. Although the rate of complications was high, 73 % had a successful reinsertion of the autologous graft at a low cost. We feel this result justifies the continued use of cryopreserved bone flaps.

## Background

The use of decompressive craniectomy (DC) with duraplasty for life-threating raised intracranial pressure (ICP) in traumatic brain injury (TBI) and stroke patients is becoming widespread. The clinical benefit of DC is reasonably well documented for selected stroke patients but remains a subject of discussion for TBI patients [1, 2]. The procedure is later followed by a cranioplasty procedure (CP) with reinsertion of the cryopreserved autologous bone flap or a synthetic implant. Synthetic implants are generally used in cases of severe comminution of the skull bone or open injuries with contamination of the surgical field. Although the surgery is rather straightforward in most cases, there is a growing body of evidence suggesting high complication rates, including surgical site infection (SSI) and bone flap resorption (BFR) [3–7]. This study is a retrospective, single-institution survey of all stroke and TBI patients undergoing acute DC and subsequent CP over a 10-year period with a focus on risk factors for post-operative complications following CP. Identifying such risk factors is important for treatment planning in order to reduce the risk of complications in this patient group.

## Methods

All stroke and TBI patients with refractory raised ICP operated with an acute DC between January 1, 2003 and June 30, 2013 at Oslo University Hospital, Ullevål were included. The group of patients with stroke included subarachnoid hemorrhages, cerebral hemorrhages and media infarctions. Patients undergoing craniectomies for tumors, post-operative infections or complex fractures without raised ICP were excluded. All procedures were performed as standard unilateral hemispheric craniectomies with duraplasty, intravenous antibiotics and post-operative subcutaneous drains. The bone flaps were cleaned of soft tissue, covered in two sterile layers and stored within a hard box at -80 °C without the application of local antibiotics or autoclaving. The bone flaps were registered as intact or fragmented. Fragmentation of the removed graft into two or more parts was caused either by fractures or by re-operation with extension of a previous craniotomy. The various bony parts were fixed with plates and screws either at the time of DC or during CP. CP was performed with reinsertion of the thawed autologous bone flap or a synthetic implant, applying per-operative intravenous antibiotics and post-operative subcutaneous drains. Synthetic implants were used in cases were the harvested bone flap had to be discarded due to severe comminution or contamination. Over the 10-year period, a total of 4 different constructs were used in the department (CustomBone®, Finceramica, Italy; HTR-PMMA®, Biomet, USA; Medpor®, Stryker, USA and Zimmer, Palacos® bone cement, USA). All DC and subsequent CP procedures were identified, and a database was created with relevant variables, including post-operative complications (Table 1). Hydrocephalus and epileptic seizures were not registered as complications of CP, as these conditions were considered to be associated with the preceding pathology. Functional outcome was estimated using the Glasgow Outcome Scale (GOS) at time of the CP. Patients developing unacceptable cosmesis or softening of the autologous bone flaps were examined with CT scans. BFR was defined as the development of osteolytic lesions in the autologous graft affecting both the internal and external tabula and without any clinical or biochemical signs of infection.

**Table 1:** Recorded variables

Patient characteristics	Age, gender, co-morbidity^a^, GCS^b^, GOS, indication for DC, VP-shunt, 30-day mortality
Operative measurements	Operating time (CP), surgery prior to DC, implant type, time between DC and CP, fragmentation of bone flap
Complications	SSI, wound dehiscence, BFR, hematoma, implant displacement

^a^Previously known psyciatric illness, substance abuse, diabetes, heart, lung or kidney disease

^b^ *GCS* Glasgow Coma Scale (last recorded before DC), *GOS* Glasgow Outcome Scale, *DC* decompressive craniectomy, *CP* cranioplasty, *SSI* surgical site infection, *BFR* bone flap resorption

### Statistical analysis

Statistical analysis was performed using SPSS Statistics, version 21 for Windows (IBM, Armonk, New York, 2012). Statistical significance was defined as a *p* value < 0.05. The Mann-Whitney U test was used for continuous variables without normal distribution. Continuous variables are reported as median values with range (min-max), while categorical data are reported as frequencies and percentages. To identify possible risk factors for SSI and BFR univariable and multivariable logistic regression analyses were used. Potential predictors of complications that were statistically significant or borderline significant in the univariable model were included in the multivariable analysis but limited to 4 variables due to the relatively low number of patients in the study. The Glasgow Outcome Scale (GOS) was modeled as a continuous variable in the regression analysis.

### Ethical considerations

This study was approved by the local ethics board as a quality assessment investigation.

## Results

**Fig. 1:**
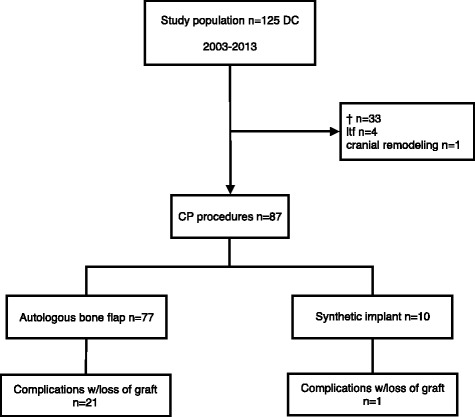
Flow chart of patients in the retrospective study. † died, *ltf* lost to follow-up, *DC* decompressive craniectomy, *CP* cranioplasty

A total of 125 patients underwent an acute DC during the specified time period. The procedure was the initial surgical intervention in 63 patients, while 28 patients underwent a prior craniotomy. In the remaining cases, ICP-measuring probes with or without extraventricular CSF drainage were initially placed. Trauma was the most common indication for DC (103/125 patients). Thirty-day mortality was 22 % (28/125 patients), and an additional 5 patients died before a CP could be performed (Fig. 1). Four patients were lost to follow-up because of transfer to other hospitals outside the region. One infant underwent a local cranial vault remodeling as a CP and was excluded from this series. The median age of the remaining 87 patients was 31 years (1-65 years). Autologous bone grafts were used in 77 patients while 10 received a synthetic implant. A median operating time of 85 (35-390) minutes during CP was found. Overall, median time between DC and CP was 74 (19-353) days. The results are summarized in Fig. 1 and Table 2.

**Table 2:** Patient characteristics and results of all CP procedures

Variable	CP
Median age (years)	31 (1-64)
Male-n (%)	54 (62)
Preceding pathology-n (%)	
- Trauma	74 (85)
- Stroke	13 (15)
Surgery prior to DC-n (%)	
- None	40 (46)
- Craniotomy	23 (26)
- ICP and/or EVD	24 (28)
Implant - Autologous-n (%)	77 (89)
- Synthetic-n (%)	10 (11)
Median operating time (min) (range)	85 (35-390)
GOS level-n (%)	2 = 16 (18)
	3 = 30 (35)
	4 = 20 (23)
	5 = 21 (24)
Complications-n (%)	
- Wound dehiscence	1 (1.1)
- Implant dislodgement	3 (3.4)
- Intracranial hematoma	5 (5.7)
- SSI	8 (9.2)
- BFR (of 71 patients)	14 (19.7)

*DC* decompressive craniectomy, *CP* cranioplasty, *ICP* intracranial pressure, *EVD* External ventricular drainage, *GOS* Glasgow Outcome Scale, *BFR* bone flap fragmentation, *SSI* surgical site infection

Median time of follow-up for all patients was 9.9 months. A post-operative CT scan was performed after the CP and before discharge in all patients. Ten patients were not registered with further post-operative clinical or radiological controls at our institution and assumed to be handled by their local hospital. The remaining patients underwent clinical controls with or without CT scanning at the physicians discretion.

### Complications

One or more complications were observed in a total of 31/87 (36 %) patients (Table 2). The rate of complications was equal in trauma and non-trauma patients. Surgical re-intervention was performed for wound dehiscence (1/87 patients), implant dislodgement (3/87 patients) and intracranial hematomas (5/87 patients). SSI with subsequent removal of the skull implant occurred in 8/87 (9.2 %) cases, of which one was synthetic. A microbiological culture was positive in all, with 5 infections caused by *Staph. aureus* and 3 by *Staph. epidermis*.

Of the 77 patients with autologous bone flaps, 6 had the graft removed and disposed of earlier than 4 weeks, due to either infection (4 cases) or a post-operative hematoma with per-operative brain edema (2 cases). Thus, 71 patients were eligible for long-term observation with a median time of follow-up of 9.4 months. BFR was observed in 14 of these patients, of whom 12 were considered to have clinically significant and/or progressive development of graft necrosis necessitating revision surgery with a synthetic implant. In the remaining two cases of BFR, further surgery was not performed because bone resorption was only affecting a minor portion of the implants and did not progress on repeated CT examinations. Both of these patients have been followed for more than 5 years after CP.

To identify risk factors associated with the development of SSI and BFR, binary, logistic regression analyses were performed using univariable and multivariable modeling. No significant predictors of SSI were found, and the rate of complications was not affected by the choice of implant material (autologous versus synthetic) or initial pathology (trauma versus stroke). Fragmentation of the bone flap, storage time of the bone flap and GOS at time of CP were significantly associated with BFR in the univariable analysis (Table 3). Age was borderline significant (*p* = 0.065) in the univariable analysis and significant in the multivariable analysis, indicating a negative association between increasing age and risk of BFR. Of the 5 patients aged 15 years or younger, all sustained a head trauma and underwent an acute DC. Of these patients, 4 developed BFR even though bone flap fragmentation was observed in only 1 of these cases.

**Table 3:** Results of univariable and multivariable logistic regression analyses of risk factors for BFR in autologous bone flaps

Variable	No BFR (n = 57)	BFR (n = 14)	Univariable OR (95 % CI)	Multivariable OR (95 % CI)
			p-value	p-value
Median age (range) (yrs)	38 (14-65)	26.5 (1.0-60)	0.96 (0.92-1.00)	0.94 (0.88-1.0)
			0.065	0.04
Gender - n male	36	9	0.95 (0.28-3.22)	
			0.94	
Preceding pathology* -n				
- Trauma	48	10	0.47 (0.12-1.83)	
- Stroke	9	4	0.28	
Surgery prior to DC	31/26	8/6	1.03 (0.63-1.68)	
(yes/no)			0.92	
Fragmentation -n	8	7	6.13 (1.69-22.2)	14.3 (2.26-89)
			0.006	0.005
Median operating time (min) (range)	81.5 (35-390)	72.5 (51-164)	0.99 (0.98-1.00)	
			0.27	
GOS at time of CP				
2	14	0	2.2 (1.16-4.20)	2.55 (1.04-6.23)
3	19	4	0.02	0.04
4	14	4		
5	10	6		
Median time DC-CP (range) (days)	70 (19-171)	88 (48-353)	1.02 (1.00-1.03)	1.03 (1.00-1.04)
			0.02	0.02
Median GCS prior to DC	6	8	1.12 (0.95-1.32)	
			0.19	
Registered co-morbidity -n	13	4	1.01 (0.77-1.32)	
			0.93	
VP-shunt at time of CP -n	5	2	1.73 (0.30-10.0)	
			0.54	

*DC* decompressive craniectomy, *CP* cranioplasty, *GCS* Glasgow Coma Scale, *VP* ventriculoperitoneal, *BFR* bone flap resorption

*Trauma modeled as the reference category in the univariable analysis

Bone flap fragmentation was noted in 15 patients of whom 11 were trauma cases on which the skull bone had been fractured. In the remaining 4 cases, patients were reoperated and the previous craniotomy extended thus creating a new bone flap consisting of two pieces.

### Revision CP

A total of 22 patients experienced a complication necessitating a revision cranioplasty with a syntethic implant (Fig. 1). The indications were BFR (12), SSI (8) or post-operative hematomas (2). A new post-operative infection occurred in four patients (18 %). A post-operative hematoma requiring re-operation was observed in 2 patients (9.1 %), and 1 patient underwent revision surgery due to wound dehiscence. The rate of complications was not dependent on the type of synthetic implant.

## Discussion

CP ensures cosmesis and protection of the underlying brain and may even improve post-operative neurological status [8–10]. Reinsertion of the previously harvested autologous bone flap is the method of choice for most institutions because of its low cost and excellent anatomical fit. Various allografts and synthetic materials have also been tried, with methyl methacrylate (MMA) and polyetheretherketone (PEEK) being the most commonly applied today [11, 12].

A number of studies have suggested high complication rates related to CP [3, 5–7]. In addition to post-operative extra- and intracranial hematomas, SSI and BFR are the most frequently described adverse events in the literature. An infected or necrotic bone flap will usually have to be explanted and replaced with a synthetic implant, adding additional insult to patients who are recovering from their preceding pathology. The identification of predictive parameters for these post-operative complications is therefore valuable to clinicians in an attempt to improve patient outcome.

### Wound dehiscence, implant dislodgement and post-operative hematomas

Wound dehiscence without concurrent infection was observed in 1.1 % of the patients, necessitating only a minor skin revision. Implant dislodgement occurred in 3.4 % of the patients due to loosening of the fixation material. In 2 of these patients, the implant displacement was obvious on the immediate post-operative CT scan, and a reoperation was performed within the same day, while the third patient developed gradual loosening of the bone graft over approximately two years before replacement with a synthetic implant. Post-operative hematomas requiring surgical evacuation after CP occur in 2-12 % of cases in the literature [13–15]. In this series, 5.7 % of the patients experienced hemorrhages, all of which were extradural. Though causing a significant deterioration in the neurological status before evacuation, all patients had regained their initial pre-operative functional level at time of discharge.

### SSI

SSI was recorded in 9.2 % of patients, which is consistent with the reported rates of infection, 7-22 % [3, 13, 14, 16]. We failed to identify any risk factors associated with SSI in the regression analysis. Im et al. reported a non-significant trend toward increased risk of post-operative infection with early CP, and the work of Thavarajah et al. indicates that the procedure should be postponed as late as 6 months to minimize the risk [17, 18]. However, in a meta-analysis of 18 articles, Yadla et al. found no difference in the infection rates of early (<3 months) versus delayed (> 3 months) surgery, and later studies showed the same [19–22]. Likewise, the choice of graft material (autologous versus synthetic implants) and indications for DC (trauma versus non-trauma) do not seem to influence the rate of infections [14, 23].

### BFR

The potential risk of autologous bone flap resorption is well known, but the reported rates vary greatly in the literature, ranging from 1.4 % to 22.8 % [4, 14, 16, 22]. Of the 87 patients undergoing CP, 71 patients were eligible for long-term follow-up with regard to graft necrosis. Of these patients, 19.7 % developed clinical and radiological signs of bone necrosis with softening and osteolysis of the graft. In the multivariable logistic regression analysis, young age, fragmentation of the explanted bone graft and delayed time of CP were independent and significant predictors of BFR. Surprisingly, a high GOS at the time of CP was also associated with risk of BFR in the uni- and multivariable models (Table 3). However, we believe this result implies a bias in our material: of the 57 patients registered without BFR, 25 % were in a permanent vegetative state (GOS 2) compared to 0 % in the BFR group (Table 3). It is possible that softening of the implanted autologous bone flap may have been undetected or ignored by the patients’ care-takers in the former group and thus not brought to our attention, as these patients were not routinely followed-up due to their low functional status.

Young age is a known risk factor of BFR, as demonstrated in studies on pediatric patients with rates of necrosis as high as 81.8 % [5, 24]. In this study, 4 out of 5 patients younger than 15 years of age developed osteolysis and underwent revisional CP with a synthetic implant. Some argue that the age factor may be related to growth of the calvaria [5]. During this phase, the pediatric skull is undergoing a dynamic process of remodeling that includes osteoclastic mechanisms, theoretically making the autologous bone flap vulnerable to the development of osteolysis. However, experimental studies on animals demonstrate improved osteogenic capabilities in younger individuals with concurrent lower resorption rates, and the cause of this age factor is largely not understood [25].

Fragmentation of the bone flap either by trauma or extension of a pre-existing craniotomy showed a clear correlation with the risk of BFR, and this result is supported by the findings of others [16, 24, 26]. Cryopreservation renders the autologous bone flap free of viable osteoblasts, and the survival of the implant is dependent on the ingrowth of new blood vessels from the bony edges, periostal layer and dura after reimplantation [25]. Bone fractures are known to disrupt blood circulation and potentially disturb angiogenesis, which precludes osteogenesis [27]. We theorize that the physical gap itself created by a fracture line or craniotome in the bone flap may also interfere with later angiogenesis and the survival of the graft.

Though extended storage in the freezer was found to increase the risk of BFR in our study, Schuss et al. showed early CP (≤ 2 months) to increase the risk [26]. Other studies fail to demonstrate an association between CP timing and risk of BFR at all [4, 16, 21]. In general, the autologous bone flap harvested at the time of DC may be stored in a freezer or in a subcutaneous pocket on the abdomen or thigh. Subcutaneous banking has the advantage of potentially supplying nutrition to the autologous graft [28–30]. In fact, there are examples of new bone formation within the bone flap with this method [30]. The low rates of BFR with subcutaneous storage are encouraging, even though the disadvantage of a second incision and operating field is obvious. However, there seems to be remodeling of the bone flap during intracorporal storage, with gradual loss of volume. This remodeling complicates the later CP with the need for additional foreign materials to fill cranial defects [28, 30].

### Synthetic implants

Ten patients received a synthetic implant during primary CP due to either contamination or severe comminution of the original bone flap. No difference was noted among the 4 various synthetic implants with regard to cosmesis or recorded complications, but the number of cases is too small for statistical analysis. One patient developed a post-operative infection and had the implant removed. Synthetic implants do not develop resorption but are prone to fractures and carry a significantly greater cost [31]. Though the total complication rate in this patient group was only 10 % compared to 39 % for patients receiving autologous bone flaps, the number of patients in the former group is too small to draw any conclusions.

### Revision CP

Revision CP was performed in 22 patients, of whom 7 had post-operative complications necessitating further surgery. Five required more than one reoperation due to wound dehiscence or SSI, emphasizing the susceptibility to surgically related adverse events in this patient group.

## Conclusions

CP following acute DC carries a high risk of post-operative complications. In this study, 31 patients (36 %) experienced one or more adverse events leading to loss of the primary implant in 22 of these cases (25 %). SSI and BFR were the most common complications of which risk factors could be identified for BFR only. A primary synthetic implant may be considered in cases with fragmented bone flaps, delayed time to CP or in pediatric patients (< 15 years). Time from DC to CP was statistically significant with regard to risk of BFR in our study, which contradicts the findings of most other studies. We recommend that patients undergoing CP should be clinically monitored with a routine follow-up consultation due to the high risk of complications that may appear months after surgery. Although 21 of 77 patients experienced a complication leading to loss of the autologous bone flap, the remaining 56 patients (73 %) had a successful reinsertion with good cosmesis and a low cost. We feel this result justifies the continued use of cryopreserved autologous bone flaps.

## Abbreviations

BFR: bone flap resorption
CP: cranioplasty procedure
CSF: cerebrospinal fluid
DC: decompressive craniectomy
EVD: extraventricular drainage
ICP: intracranial pressure
SSI: surgical site infection
TBI: traumatic brain injury
